# Combining linkage data sets for meta-analysis and mega-analysis: the GAW15 rheumatoid arthritis data set

**DOI:** 10.1186/1753-6561-1-s1-s104

**Published:** 2007-12-18

**Authors:** Ricardo Segurado, Marian L Hamshere, Beate Glaser, Ivan Nikolov, Valentina Moskvina, Peter A Holmans

**Affiliations:** 1 Biostatistics and Bioinformatics Unit and Department of Psychological Medicine, Cardiff University, School of Medicine, Heath Park, Cardiff CF14 4XN, UK

## Abstract

We have used the genome-wide marker genotypes from Genetic Analysis Workshop 15 Problem 2 to explore joint evidence for genetic linkage to rheumatoid arthritis across several samples. The data consisted of four high-density genome scans on samples selected for rheumatoid arthritis. We cleaned the data, removed intermarker linkage disequilibrium, and assembled the samples onto a common genetic map using genome sequence positions as a reference for map interpolation. The individual studies were combined first at the genotype level (mega-analysis) prior to a multipoint linkage analysis on the combined sample, and second using the genome scan meta-analysis method after linkage analysis of each sample. The two approaches were compared, and give strong support to the *HLA *locus on chromosome 6 as a susceptibility locus. Other regions of interest include loci on chromosomes 11, 2, and 12.

## Background

Problem 2 of Genetic Analysis Workshop 15 (GAW15) includes genome-wide genotyping of marker sets for linkage studies in rheumatoid arthritis (RA). Four research groups contributed sets of markers across the genome genotyped in four independent pedigree samples. NARAC (North American Rheumatoid Arthritis Consortium) held by far the largest sample, consisting of multiplex families genotyped for 10-cM linkage mapping set and a panel of single-nucleotide polymorphisms (SNPs) genotyped by Illumina. A Canadian group provided pedigrees genotyped for the same Illumina marker panel as well as a dense 100 k Affymetrix SNP map. ECRAF (European Consortium on Rheumatoid Arthritis Families) genotyped a dense microsatellite panel. There was also a United Kingdom (UK) data set, comprising both microsatellite and SNP markers with a two-stage design.

The diversity of these samples and marker maps presents a complex problem to anyone seeking to merge them in order to achieve the greater power of a combined sample. In particular, we were interested in judging whether there is a "best" way to merge marker maps, or if available information would force one particular solution. We combined the data by placing markers on a common genetic map and performing linkage analysis on the whole sample jointly (e.g., McQueen et al. [1]), which we termed a "mega-analysis". A meta-analysis technique, the genome scan meta-analysis (GSMA) [2], was used as a comparison for ease of methodology. This method involves the division of the genome into a specific number of fixed-width "bins", which are then each ranked within a study according to the evidence for linkage within the bin (LOD score or *p*-value), with a concomitant loss of spatial accuracy. The GSMA statistic is calculated for each bin as the mean rank across studies, with significance levels determined by permutation of the observed ranks within each study.

We were also interested in determining the evidence for linkage across the genome in each of the four samples compared with mega-analysis and meta-analysis. The NARAC sample is considerably larger than the others (1637 affected individuals versus 118, 187, and 332 in the Canadian, ECRAF, and UK samples, respectively), and might be expected to outweigh the others in any joint analysis.

## Methods

### Marker maps

Four data sets were analyzed for autosomal genetic linkage to RA (Table 1). The Canadian Affymetrix genotypes were excluded owing to difficulties in placing markers and time considerations. In cases in which several marker sets were available in the same sample, the most informative was selected as detailed below.

**Table 1:** Descriptions of the data sets

Sample	Markers	No. Pedigrees (No. affected individuals)	No. markers	Map
UK	SNPs	157 (332)	2473	cM map 1
NARAC	Illumina SNPs	725 (1637)	2364	base-pair position
ECRAF	Microsatellites	88 (187)	872	cM map 2
Canada	Illumina SNPs	59 (118)	2364	base-pair position

We assembled all markers from all studies onto a common centimorgan (cM) genetic map using a procedure described in Hamshere et al. [3]. Markers that could not be positioned were removed from the analysis. We were provided with a genetic and physical sequence (RefSeq) alignment of the NARAC and ECRAF microsatellite markers. The base-pair position provided for Illumina SNPs (NARAC and Canada) did not correspond to the physical map, and so these markers were queried against the NCBI database to obtain updated positions comparable with the NARAC and ECRAF microsatellite positions.

The UK microsatellite positions were judged to be on the same map as NARAC, on the basis of 19 (out of 20) microsatellites common to both sets, which had identical centimorgan positions. Because the UK SNP marker map was of unknown provenance and the marker names cryptic, it was assumed to be comparable if not identical to the UK microsatellite map.

Because all other marker sets could be positioned relative to the NARAC/UK genetic map with a minimum number of assumptions, this was used as a standard map, and the NARAC microsatellites were used as reference markers (RMs). NCBI base-pair positioning permitted the interpolation of all other markers into each RM interval. Markers positioned outside of this standard map on each chromosome were removed from the analyses to prevent the possibility of negative map positions or overinflated chromosomal lengths. A total of 517 markers were removed from the NARAC and Canada SNP maps, and 74 markers from the ECRAF microsatellite map. A high level of correlation was observed between the original map and our standard map (interpolated on the basis of sequence positions) for the ECRAF microsatellites (Pearson *r *> 0.99). Of note are three markers which differed considerably in genetic position between the two maps: D9S144, D12S43, and D16S289 were originally positioned at 0 cM, and shifted by between 20 and 100 cM in the standard map, due to their current position in the sequence database. These instances were excluded from our analysis, and should be followed up in order to be sure of the identity and location of the typed marker.

### Data cleaning

All pedigrees were examined for Mendelian inconsistencies using the PedCheck program [4]. The GRR software [5] was used to detect potential misspecification of within-family relationships or sample mix-ups, which were then removed. We screened out pedigrees containing individuals of non-Caucasian/European ancestry, where known.

### Linkage disequilibrium

Multipoint linkage disequilibrium (LD) was eliminated from each data set in order to prevent artificial inflation of the multipoint linkage statistics arising from incorrect allele frequency estimation in cases of missing parental data [6]. In the first instance, each marker map was thinned to a minimum intermarker distance of 0.5 cM. The microsatellite map was adjusted for LD as detailed by Hamshere et al. [3]. For the SNP markers, pair-wise LD in founder individuals, or the whole sample if insufficient founders were genotyped, was measured using LDMAX [7]. Marker pairs further apart than 5 cM were ignored because this was felt to be unlikely to affect the multipoint linkage statistic. A list of marker pairs in LD was compiled and one marker from the first pair was removed from the analysis, and the list was updated. The process was repeated until no marker pairs with *r*^2 ^> 0.05 remained. We removed 7683 of the UK SNPs, 2546 of the NARAC and Canada SNPs, and 88 of the ECRAF microsatellites.

### Linkage analysis

Multipoint inkage analysis of the RA binary trait was performed on a 2-cM grid using MERLIN [8], with the 'npl' and 'exp' options. The average information content was higher for the UK SNP maps than for the microsatellite data. The NARAC SNP markers were also typed for a larger sample than the microsatellites. We used the SNP genotypes, rather than the microsatellite genotypes, for these two samples.

### Meta-analysis

A GSMA was performed as described previously [2,9]. The recommended bin boundaries for 120 30-cM bins were used in order to permit direct comparisons with previous studies. The bin-boundary markers were interpolated onto the NARAC genetic map, and all markers were 'binned' by reference to their positions. The maximum LOD score in each bin was used by the GSMA program to derive ranks for each bin, weighted by the square root of the number of affected individuals, and genome-wide significance was calculated from 10,000 permutations.

## Results and discussion

The maximum LOD score on each chromosome from the four individual samples are presented in Table 2 alongside the results of the mega-analysis. Chromosome 6 showed the highest LOD (20.71) at 46 cM in the mega-analysis, as well as the highest LOD in each of the individual screens (with the exception of Canada). It was the only locus with LOD > 0.5 observed in the UK sample.

**Table 2:** Maximum linkage score of each chromosome in the four genome screens, and combined analysis

	Mega-analysis		UK		NARAC		ECRAF		Canada	
					
Chr	LOD	cM	LOD	cM	LOD	cM	LOD	cM	LOD	cM
1	**1.02**^a^	240	0.44	156	**1.28**	234	**1.69**	28	0.57	272
2	**2.01**	194	0.07	238	**3.16**	192	**2.03**	86	0.81	104
3	0.17	64	0.24	118	0.43	78	0.69	146	0.4	54
4	**1.83**	110	0.01	64	**2.77**	110	**1.16**	2	0.25	0
5	**1.42**	26	0.04	186	**3.35**	26	0.85	164	0.18	186
6	**20.71**	46	**2.85**	58	**16**	46	**2.93**	44	**1.39**	156
7	**1.66**	126	0.03	84	**2.08**	144	0.33	140	**1.93**	34
8	0.72	104	0.33	102	**1.16**	118	0.2	92	0.73	68
9	0.76	146	0.04	32	0.54	146	0.1	136	**2.12**	144
10	**1.69**	102	0.3	154	**2.46**	90	0.13	102	0.23	6
11	**2.81**	48	0.01	26	**4.02**	48	0.01	42	0.53	28
12	**1.29**	42	0.31	136	**1.55**	44	**1.5**	104	**1.16**	118
13	0.51	80	0.36	80	**1.01**	28	**2.46**	102	0.18	92
14	0.27	108	0.36	76	0.67	92	0.16	126	0.33	114
15	0.68	100	-0.01	104	0.7	100	0.21	98	0.48	104
16	**1.07**	78	0.16	20	**1.49**	68	0.57	46	0	72
17	0.85	86	0.15	82	0.99	106	0.73	6	0.58	0
18	**1.03**	76	0	112	0.96	80	0.94	100	0.37	20
19	0.88	96	0.19	86	0.43	98	0.12	56	**1.11**	102
20	0.37	88	-0.03	98	**1.1**	86	**1.9**	4	0.16	56
21	**1.37**	42	0.32	52	**1.15**	42	0.55	40	0.11	30
22	0.24	60	0.03	60	0.2	42	0.71	8	0.02	14

^a^LOD > 1 highlighted in bold.

High linkage scores were also detected on chromosomes 11 (LOD = 2.81, 48 cM), 2 (LOD = 2.01, 194 cM), and 4 (LOD = 1.83, 110 cM). Loci showing low or modest linkage with some concordance across studies included chromosomes 9 (136–146 cM), 12 (104–136 cM), and 21 (30–52 cM). Five chromosomes (3, 14, 15, 17, and 22) showed no LOD score > 1.

The results of the meta-analysis (Table 3) implicate the same region as the mega-analysis on chromosome 6 as a susceptibility locus for RA, with bin 2 achieving genome-wide significance according to the Lander and Kruglyak criteria [10], and bins 1 and 3, genome-wide suggestive linkage. Interestingly, there may be some evidence for a second locus on chromosome 6. Meta-analysis of bin 5 (centered at ~150 cM) shows low/modest peaks in all the samples, and covers the location of the highest peak on the chromosome in the Canadian sample. The mega-analysis showed a LOD score peak of 2.01 at 148 cM on this chromosome.

**Table 3:** Bins with nominally significant GSMA results

Bin (chr. bin)	Position (cM)^a^	Summed rank *p*-value^b^	Ordered rank *p*-value
**6.2**^c^	28.4–64.8	**1.67 × 10^-6^**^d^	**2.0 × 10^-4^**
**6.3**	64.8–99.7	**0.0005**^e^	**0.0016**
**6.1**	0.0–28.4	**0.0017**^e^	**0.0010**
6.5	131.1–166.5	0.0152	0.0932
6.4	99.7–131.1	0.0205	0.0786
2.8	176.0–208.0	0.0270	0.0820
12.2	24.4–51.4	0.0477	0.3382
2.6	128.0–156.0	0.0487	0.1992

^a^cM position according to the NARAC map.

^b^SR *p*-value < 0.05

^c^Bold type indicates bins with both SR and OR *p*-values < 0.05.

^d^Genome-wide significant

^e^Genome-wide suggestive

Three of these samples (NARAC, ECRAF, UK) have been used in previous meta-analyses [11,12]. As in those analyses, we find our most significant results on chromosome 6p, and nominal significance on chromosomes 12p and 2q. Discrepancies such as on chromosomes 8p and 1q may be attributable to differences in the samples (we added the Canadian data set and updated versions of the other data sets; we removed non-Caucasian pedigrees) and markers (where possible we used SNP markers rather than microsatellites; we ensured LD was removed prior to linkage analysis).

Several of these bins span candidate gene loci, including the *HLA-DRB1 *locus in bin 6.2. *CTLA*, which may be a susceptibility locus for RA [13] is located in bin 2.8, the second and third most significant chromosome in the meta- and mega-analysis, respectively. The *PTPN22 *locus maps to bin 1.6, which was the 25^th ^highest ranked bin in the meta-analysis, and was not significant. This locus did not feature in any of the individual analyses, although the mega-analysis shows a small peak over the gene locus at 156 cM, with a maximum score of 0.99 (nominal *p *= 0.02).

The conduct of our study was largely focused on the details of combining samples for mega-analysis in a rigorous fashion, and we conclude that the process adopted will often be dictated by the characteristics of the samples, and the information available on each. Researchers should follow common sense in including the data that provide the maximum information for each sample. The use of commercial high-density SNP maps, and the full-genome physical maps have made this process easier and probably more accurate than in the recent past. Meta-analysis techniques such as GSMA are more straightforward to perform than a mega-analysis, since they do not require access to the genotype data. Both the meta- and mega-analyses detect the strong linkage to chromosome 6. In addition, regions on chromosomes 2 (194 cM) and 12 (42 cM) are highlighted by both analyses. However, the mega-analysis showed evidence for linkage to chromosomes 10q and 11q, which was not observed in the GSMA. This is because chromosomes 2 and 12 show consistent linkage evidence across the four samples, whereas the linkages of chromosomes 10q and 11q are due almost entirely to high LOD scores from the NARAC sample, with negligible linkage evidence in the other three samples. The GSMA assigns significance to regions where linkage statistics are consistently highly-ranked across studies, but does not take into account the magnitude of the linkage statistics. Thus, it will not detect linkages based on a very high linkage statistic from one sample.

## Conclusion

This is the largest combined sample analyzed so far for linkage to RA. Both meta- and mega-analysis detected a highly significant linkage to chromosome 6p, with weaker linkages to chromosomes 6q, 2q, and 12p. In each of these regions there was consistent linkage evidence across the four samples. The mega-analysis also detected linkage to chromosomes 10q and 11q. In these regions, the linkage evidence came almost entirely from the NARAC sample, so they were not picked up by the meta-analysis. When performing an analysis of combined samples, it is important to ensure that marker maps are compatible, and that as many genotyping errors as possible are removed. It is also important to keep inter-marker LD to a minimum.

## Competing interests

The author(s) declare that they have no competing interests.
